# Population genetic structure of *Ascaridia galli* re-emerging in non-caged laying hens

**DOI:** 10.1186/1756-3305-5-97

**Published:** 2012-05-20

**Authors:** Johan Höglund, David A Morrison, Annie Engström, Peter Nejsum, Désirée S Jansson

**Affiliations:** 1 Department of Biomedical Sciences and Veterinary Public Health, Swedish University of Agricultural Sciences, Section for Parasitology, P.O. 7028, Uppsala, SE-750 07, Sweden; 2 Department of Veterinary Disease Biology, Danish Centre for Experimental Parasitology, Faculty of Life Sciences, University of Copenhagen, Copenhagen, Denmark; 3 Department of Animal Health and Antimicrobial Strategies, National Veterinary Institute (SVA), Uppsala, SE-751 89, Sweden

**Keywords:** AFLP, *Ascaridia galli*, Nematoda, Parasite infection, Population genetics, Network analysis

## Abstract

**Background:**

The poultry roundworm *Ascaridia galli* has reappeared in hens kept for egg production in Sweden after having been almost absent a decade ago. Today this is a frequent intestinal nematode parasite in non-caged laying hens. The aim of this study was to investigate the genetic diversity (F_st_) in *A*. *galli* collected from different poultry production sites in southern Sweden, to identify possible common routes of colonization.

**Methods:**

Adult parasites (n = 153) from 10 farms, including both broiler breeder parents and laying hens, were investigated by amplified restriction fragment length polymorphism analysis (AFLP). Worms from a Danish laying hen farm were also included for comparison. Most of the farms were represented by worms from a single host, but on two farms multiple samples from different hosts were assessed in order to study flock variation.

**Results:**

A total of 97 fragments (loci) were amplified among which 81% were variable alleles. The average genetic diversity was 0.13 (range = 0.09-0.38), which is comparable to other AFLP studies on nematodes of human and veterinary importance. Within-farm variation showed that worms harboured by a single hen in a flock covered most of the *A. galli* genetic variation within the same flock (F_st_ = 0.01 and 0.03 for two farms). Between-farm analysis showed a moderate population genetic structure (F_st_ = 0.13), along with a low mutational rate but high gene flow between different farms, and absence of strong genetic selection. Network analysis showed repeated genetic patterns among the farms, with most worms on each farm clustering together as supported by high re-allocation rates.

**Conclusions:**

The investigated *A*. *galli* populations were not strongly differentiated, indicating that they have undergone a genetic bottlenecking and subsequent drift. This supports the view that the investigated farms have been recently colonized, and that new flocks are reinfected upon arrival with a stationary infection.

## Background

Organisms vary genetically as a reflection of their evolutionary history and, thus information about the population genetic structure is basic to the understanding of biodiversity. Quantifiable components of this structure include: genetic diversity, population hierarchical structure, population mutation rate, rate of gene flow, and selective neutrality [1]. Parasitic nematodes of livestock are no exception to this universal biological rule. During recent decades an ever-increasing amount of genetic data have been generated from populations of nematode parasites to elucidate micro-evolutionary processes [2-4].

However, unlike most free-living organisms, genomic variation of parasites is not only influenced by their own reproductive and transmission patterns but also by host genetics and behavior (e.g. migration) [2]. In the case of livestock parasites, transfer between production sites through active host movement and/or by contaminated fomites certainly plays an important role. Access to genetic approaches opens up opportunities to trace how nematode infections are transmitted both within and between different host populations [3]. Thus, by using genetic markers we can understand and depict geographical movements of parasitic nematodes, for example as a result of changes in animal husbandry. Data on the population diversity and structure may also be valuable for the tracing of the escalating spread of drug resistance among parasitic nematodes of livestock, for a review: [5]. Direct sequencing of PCR-amplified mitochondrial DNA is the most frequently used molecular-based strategy, which has been used to infer the population genetic structure and diversity of a number of animal nematodes, for a review: [4]. However, single gene analysis may lead to wrong conclusions [6]. Applying whole-genome methods such as AFLP (amplified fragment length polymorphism) overcomes this problem at a reasonable cost by including genetic information from many loci randomly distributed throughout the entire genome. AFLP is a very sensitive and a highly reproducable PCR-based genomic fingerprinting technique, which generates variable multilocus dominant markers [7]. As a result, AFLP has become widely used for the identification of genetic variation in studies of organisms with complicated genomes such as in plants and fungi [8]. Currently, few AFLP based studies have been conducted on nematodes of domesticated animals: *Dictyocaulus viviparus*, a trichstrongylid lungworm of ungulates [9], *Ascaris suum*, an Ascarid intestinal parasite of pigs [10], and *Haemonchus contortus*, a trichostrongylid abomasal parasite of ruminants [11]; as well as for *Necator americanus*, a strongylid intestinal nematode of humans [12].

By the end of 2004 the housing system for almost all Swedish commercial laying hens had been changed from conventional battery cages to furnished cages, indoor litter-based housing systems or free-range production, in order to improve bird welfare. In non-caged hens, this change was accompanied by a rapid increase of roundworm infections, especially *Ascaridia galli*[13]. The source of these nematodes and routes of transmission between farms have not been clearly identified, but previous studies have suggested indirect transmission between farms rather than introduction via infected replacement pullets [13,14]. To explore this further, more detailed studies based on the genetic relationships between worms collected from different flocks are warranted. In this study, we investigated the genetic diversity and structure of *A. galli* within and between flocks on different farms using AFLP. The overall aim was to quantify the *A*. *galli* population diversity and structure, and to compare the results with what is known about the recent reapperance of *A*. *galli* in the Swedish egg industry.

## Methods

### Study design

**Figure 1:**
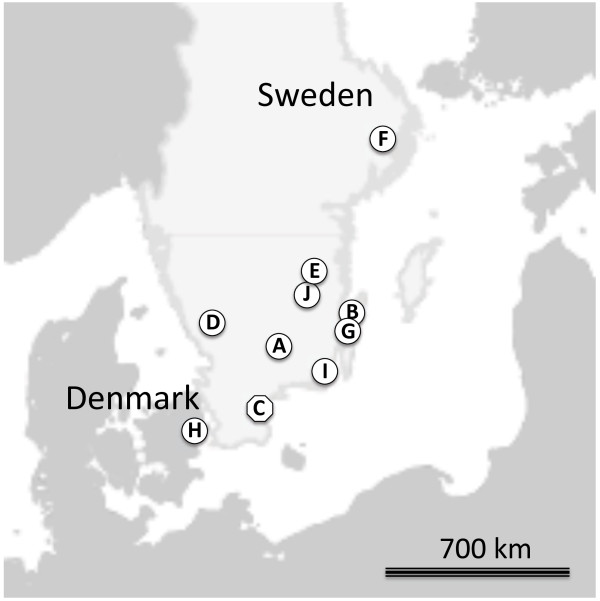
Map showing the geographical location of the sampled farms.

AFLP genetic fingerprints were obtained from adult *A. galli* from laying hens and in one population from broiler breeder parents (Table 1), basically using the same methodology as described by Höglund et al. [9]. The worms from broiler breeder and laying hens were collected either at a slaughterhouse or from birds submitted the National Veterinary Institute (SVA) for routine diagnostic necropsies. The laying hens flocks represented three hybrids and originated from different pullet breeder farms except for two flocks, which came from the same farm but from different pullet flocks raised 7 months apart. All farms included in the study were from southern Sweden (Figure 1). However, reference material from a geographically and otherwise separate organic laying hen farm in Denmark was also included.

**Table 1:** Location and characteristics of the ten sample farms

	Sampling date	Chicken category	Housing system	County / Country	Code	Number of hens
A	09-05-19	Laying hens	Aviary-indoor	Jönköping	Jonk	6
B	09-06-08	Laying hens	Aviary-indoor	Kalmar	Kal1	1
C	09-06-16	Broiler breeders	Litter indoor	Skåne	Skan	6
D	09-06-25	Laying hens	Aviary-indoor	Halland	Hall	1
E	09-07-04	Laying hens	Aviary-indoor	Östergötland	Ost1	1
F	09-07-06	Laying hens	Litter indoor	Stockholm	Stoc	1
G	09-07-29	Laying hens	Litter indoor	Kalmar	Kal2	1
H	09-08-11	Laying hens	Organic	Denmark	Danm	10
I	09-08-21	Laying hens	Free-range	Blekinge	Blek	1
J	09-08-25	Laying hens	Organic	Östergötland	Ost2	1

From most farms, 10 roundworms were collected from an individual hen (Table 1). However, from one farm a total of 56 roundworms from six different hens were collected (8–10 worms per bird), in order to compare the genetic variation between worms from different birds in the same flock; and this were treated as a separate dataset for analysis. Furthermore, the sample from Denmark, which was represented by 2 worms from each of 9 birds, were also analysed as a separate dataset. Amplified Fragment Length Polymorphism Genomic DNA was prepared using QIAamp DNA Mini Kit (Qiagen) according to the tissue protocol provided by the manufacturer, and stored at 4°C. AFLP profiles were generated as described by Applied Biosystems (ABI, Foster City, CA) in the Plant Mapping Protocol. All reagents were supplied in the AFLPTM Plant Mapping Kit except the restriction enzymes (*Eco*RI and *Mse*I) and the T4 DNA ligase, which were from New England Biolabs. The restriction enzymes were used together with two classes of selective primers with three additional nucleotides at their 3′-end. PCR amplifications were performed in a PTC-200 Peltier Thermal Cycler (MJ Research) with fluorescent labeled *Eco*RI primers (5′-end), followed by fragment detection on a Genetic Analyzer (ABI 3100). Data were analysed using GeneMapper software (version 3.7), in which the obtained peaks were sized and converted to binary characters (i.e. 0 and 1 alleles). The AFLP procedure requires a decision to be made about the cut-off level for when to score an AFLP peak from a given locus as present or absent: a low threshold will produce too much ‘noise’ while a high level will reject too much real genetic variation. There are few established criteria for this [15-17], and none of these were directly applicable for our purposes. Therefore, we heuristically chose a cut-off value based on a combination of: (i) minimizing the observed differences among replicate AFLP analyses (from a single worm), and (ii) maximizing the success of re-allocating the worms to their farm of origin. For this purpose, two and four replicate AFLP analyses were performed for two of the isolates from the farms in Jönköping and Denmark, respectively.

### Data analyses

There are many methods available for quantifying various aspects of population genetics [18]. Here, we have established a protocol for the analysis of AFLP data in nematodes, based on a variety of different computer programs. All of the analyses consider the AFLP fragments as diploid dominant markers, assuming that each fragment represents a single locus with two alleles, where fragment presence represents the dominant allele. Hardy-Weinberg equilibrium (F_is_ = 0, so that inbreeding is absent) is also assumed, as is the absence of sequence recombination. Conversion of data formats used AFLPdat (version 20.10.2010) [19]. In all cases, default value settings were used for the computer programs except as individually specified below.

Following Bonin et al. [7], the estimates of allele frequencies for each isolate used the Bayesian method with informative priors of Zhivotovsky [20]. The calculation of within-population heterozygosity (H_j_), genetic diversity (H_s_ and H_t_) and population structure (F_st_) from these estimates followed the procedures of Lynch and Milligan [21], with 10,000 randomizations used for the statistical tests. These statistics were all computed by the AFLP-Survey program (version 1.0) of Vekemans [22].

The rarity index, which is a measure of the number of rare markers in each population corresponding to the frequency down-weighed marker value proposed by Schönswetter and Tribsch [23], was calculated by the AFLPdat package. Significant deviations from expectation, either higher or lower, were tested using 10,000 randomizations. The population mutation rate (4N_μ_) was estimated using the iterative procedure described by Chakraborty and Weiss [24] based on H_j_. Gene flow between the field isolates (N_m_) was estimated using the F_st_ method described by Slatkin and Barton [25]. The Ewens-Watterson test for selective neutrality followed the method of Manly [26], with 10,000 randomizations, computed by the PopGene program (version 1.32) of Yeh et al. [27]. Pairwise genetic distances between the individual nematodes were calculated following Nei and Li [28] as modified by Felsenstein [29], using the PHYLIP computer package (version 3.69). Following Morrison [30], these distances were then visualized using a neighbor-net network, computed using the SplitsTree program (version 4.11.3) of Huson and Bryant [31].

The genetic similarity of each nematode to the other members of the same population was formally tested using the maximum-likelihood jackknife re-allocation technique described by Campbell et al. [32], based on 2000 simulated genotypes, by the AFLPop program (version 1.1) of Duchesne and Bernatchez [33].

Pairwise genetic distances between the populations were calculated following Reynolds et al. [34], using AFLP-Survey. These distances were compared to the geographical distances between the farms via Mantel tests [35], with 10,000 randomizations, using the ZT program (version 1.0) of Bonnet and van der Peer [36].

## Results

### Peak cut-off level

We evaluated cut-off levels for recognizing presence or absence of each gene fragment of 50–100 Units (U) for each sample (Table 2). A cut-off of 80 U produced the most consistent results, as it minimized the number of differences among the four replicates and maximized the re-allocation success of the combined dataset while still being close to optimal for the other two criteria (Table 2). It was thus used for all of the subsequent analyses.

**Table 2:** Assessment of cut-off levels for the presence-absence of AFLP fragments

Samples	Cut off (U)					
	50	60	70	80	90	100
	*Differences among replicates*						
Four replicates	20	11	10	5	5	5	
Two replicates	12	7	5	5	4	2	
	*Jönköping farm*						
Total no. peaks	101	88	77	68	60	55	
Variable peaks	78	65	54	46	39	35	
Re-allocation success (%)	17.9	30.4	33.9	30.4	21.4	21.4	
	*All farms*						
Total no. peaks	128	109	102	97	91	86	
Variable peaks	106	88	82	79	74	69	
Re-allocation success (%)	82.8	79.8	81.8	86.9	76.8	76.8	

### Within-chicken genetics

A total of 56 DNA samples from *A*. *galli* were analysed from the 6 chickens from Jönköping, and a total of 68 AFLP markers were generated, of which 46 were variable among the worms (Table 2). The 18 worm samples obtained from 9 chickens from the Danish farm generated a total of 69 AFLP markers, of which 50 were variable among the worms and therefore informative (Table 2).

The within-hen genetic diversity of *A*. *galli*, or heterozygosity (H_j_), which averaged across all loci excluding the non-variable ones, was in the range of 0.18–0.23 for all of the individual hens from Jönköping (Table 3). The heterozygosity was generally greater among the worms from the Danish hens (0.18–0.38; Table 3), probably due to random variation reflecting the smaller number of worms sampled per hen (2 in Denmark versus 10 in Jönköping).

**Table 3:** Within-host fragment diversity and rarity for the individual hens and farms

Sample	Number of worms	Diversity (Hj)	Rarity
	*Jönköping farm (n = 56)*			
Hen 1	10	0.1890	—	
Hen 2	10	0.1785	—	
Hen 3	9	0.1883	—	
Hen 4	10	0.2093	—	
Hen 5	9	0.2309	—	
Hen 6	8	0.1807	—	
	*Denmark farm (n = 18)*			
Hen 1	2	0.2749	—	
Hen 2	2	0.2166	—	
Hen 3	2	0.1836	—	
Hen 4	2	0.2772	—	
Hen 5	2	0.2521	—	
Hen 6	2	0.2488	—	
Hen 7	2	0.3838	—	
Hen 8	2	0.1845	—	
Hen 9	2	0.3251	—	
	*All farms (n = 99)*			
Blekinge	9	0.1417	0.8355	
Halland	10	0.0941	0.4622 *	
Jönköping	10	0.1305	0.7763	
Kalmar 1	10	0.1137	0.5806	
Kalmar 2	10	0.1103	0.5922	
Skåne	10	0.1347	0.8564	
Stockholm	10	0.1181	1.0465	
Östergötland 1	10	0.1323	0.9142	
Östergötland 2	10	0.1374	0.7990	
Denmark	10	0.1533	1.1201	

* p < 0.05 (rarity smaller than expected).

The population genetic structure (F_st_; the proportion of the total genetic variation attributed to differences between the hens) was very weak for both the Jönköping and Denmark samples (Table 4). Thus, most of the genetic variation between the nematodes is contained within individual hens, whereas little extra variation was found between hens. This result was confirmed by the low re-allocation success (Table 4), as most worms could not be correctly identified as coming from a particular hen (only 30% success for the Jönköping population). The re-allocation analysis was not performed for the Denmark sample as there were only 2 worms sampled per hen.

**Table 4:** Population genetics of the ten sampled farms and three sub-datasets

Characteristic	Jönköping farm	Denmark farm	All farms
Number of worms	56	18	99
Number of hosts	6	9	10
Total number of peaks	68	69	97
Numnber of variable peaks	46	50	79
Total diversity (H_t_)	0.1977	0.2675	0.145
Average sample diversity (H_s_)	0.1961	0.2607	0.127
Population structure (F_st_)	0.0081	0.0254	0.128
F_st_ – Sweden only	—	—	0.136
F_st_ – Non-broilers	—	—	0.124
F_st_ – Swedish non-broilers	—	—	0.133
Mutation rate (4N_μ_)	—	—	0.109
Migration rate (N_m_)	—	—	1.70
Nm – Sweden only	—	—	1.59
Re-allocation success (%)	30.4	—	86.9
Test of neutrality (% loci neutral)	76.1	74.0	74.7
Geographic correlation	—	—	0.023 *

* p = 0.497.

### Between-farm genetics

A total of 99 *A. galli* samples were analysed from the 10 flocks on separate farms (after sub-sampling the Jönköping, Skåne and Denmark samples) and a total of 97 informative AFLP markers were generated, of which 79 were variable among the worms (Table 2).

The within-farm genetic diversity (H_j_) of *A*. *galli*, was (0.09–0.15) for all of the farms, as would be predicted from the within-hen results, indicating an equivalent amount of genetic diversity among the nematodes irrespective of whether they had been obtained from a single or from multiple host animals (Table 3). Fewer rare alleles (compared to the expectation based on a random distribution) were detected on the Halland farm only (Table 3), which suggests that the alleles are randomly distributed among the other farms.

The population genetic structure (F_st_; the proportion of the total genetic variation attributed to differences between the farms) was moderate (Table 4), indicating some genetic differentiation between flocks representing different farms. The Swedish farms were analysed separately, to assess the influence of the data from the Danish samples that were separated both by geographical and other barriers, the F_st_ value remained approximately the same (Table 4). Similarly, when the worms from the broiler breeder parent hens representing a different production type than laying hens (Farm C in Skåne) were excluded from the analysis, the F_st_ value also remained approximately the same (Table 4). The population mutation rate for the nematodes (4N_μ_; the expected number of mutations under neutral evolution per locus per generation in the effective population) was only 0.11, indicating that drift dominates mutation in determining the amount of genetic variation. Furthermore, the gene flow among isolates (N_m_; the expected number of migrants per generation required to maintain the F_st_ at the observed value) was relatively high (Table 4), indicating a constant gene flow among farms. The test for neutral evolution of the variable AFLP markers was rejected in about one-quarter of the cases (Table 4).

**Figure 2:**
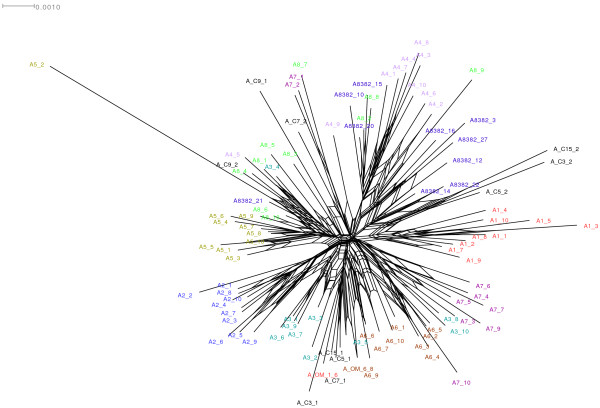
**Neighbor-net graph showing the AFLP genetic relationships among the worms sampled.** Each of the individual Ascarids is labeled with its population of origin, using the codes shown in Table 1.

The re-allocation procedure correctly assigned most (84%) of the worms to their farm of origin (Table 4). This indicates that there are recognizable patterns of genetic variation among the farms, even if those patterns are not strong (as indicated by the F_st_). This pattern of variation is most appropriately investigated and can be visualized by a phylogenetic network (Figure 2). This network shows that most of the roundworms from each of the farms form a connected cluster, along with 0–3 outliers from each flock. It is these latter individual nematodes that presumably explain the moderate F_st_ values while still allowing high re-allocation success (i.e. 8/10 worms per farm show farm-based population genetic structure while 2/10 worms do not). The only notable exception to this pattern was the sample from Denmark, where the 10 worms were scattered in 3 equal clusters, each cluster associated with a different Swedish farm (Figure 2).

The genetic differentiation among *A*. *galli* from the Swedish flocks was not correlated with the geographical distances among those farms they were representing (Table 4), suggesting absence of a simple isolation-by-distance effect.

## Discussion

We describe here for the first time the genetic diversity and population genetic structure of 154 worms of *Ascaridia galli*, a parasitic nematode of poultry, applying the whole genome fingerprinting technique AFLP. This parasite has recently received renewed attention due to its increasing prevalence in non-caged laying hens [13]. The analysed worms represented nine farms in Sweden and one in Denmark, which is separated from the others. The observed number of AFLP fragments (n = 97) and the proportion of variable loci (81%) are comparable to previous AFLP results from livestock nematodes using the same restriction enzymes and primer combinations [9,11].

In this study we first investigated the variability among genotypes of worms collected from different hosts in the same flock on a farm. This showed that Ascarid worms from a single hen are at least as genetically different from each other as are worms from different hens. As there was an apparent lack of genetic structure between worms among different hosts within a single flock, this suggests that parasites collected from a single hen can provide a representative sample of the genetic diversity contained within a particular flock. Thus, in practice *A. galli* needs only to be sampled from a single hen in order to assess the population genetic diversity of a particular flock on a farm.

This result was expected, since all birds in non-caged flocks are exposed to the same gene pool of *A. galli* eggs. However, despite similarities in the life cycle, this seems to contrast with the situation in *Ascaris* roundworms in pigs and humans, where as much as 12–20% of the genetic variation is distributed within individual hosts [10,37]. This difference is likely to be related to differing opportunities for parasite transmission. For example, pigs are usually confined to isolated pens and humans live in family groups giving local foci of transmission [38], whereas the hens in this study were housed in large flocks with thousands of individuals freely sharing the same environment. Thus, it is possible that *A. galli* populations in general are less genetically aggregated in their hosts, presumably leading to reduced inbreeding in comparison with *Ascaris*[37,39]. Irrespective of the explanation, genetic comparisons of *Ascaridia* parasites from different flocks can justifiably be based on a multiple-parasite sample collected from a single host individual in each flock.

When the parasites from different flocks on separate farms were compared, we found evidence of a relatively moderate population genetic structure (F_st_ = 0.13), and relatively little total genetic diversity (H_t_ = 0.15). However, this structure was greater than that previously reported for *Haemonchus contortus* in northern Europe (F_st_ = 0.07; [11]) but smaller than that for *Dictyocaulus viviparus* in Sweden (F_st_ = 0.39; [9]), although the heterozygosity (H_j_) was very similar (an average of 0.13, 0.20, 0.13, respectively). Thus, the lungworm *D. viviparus* shows much greater genetic differentiation between farms than do the two gastrointestinal parasites, although the levels of genetic diversity are similar. Because AFLP markers are sampled throughout the genome they are appropriate for fine scale-studies, and there is a negligible risk that the results are biased due to directional selection of a single or few gene(s) that may not be representative of the whole genome per se [7,8]. Thus, we expect this pattern to be related to differing extent of host movement between farms, or to characteristics of the parasite life history traits and/or exchange of worms in relation to production systems.

In spite of the moderate F_st_ values, there were distinct patterns of genetic differentiation among farms. The worm re-allocation success was high (87%) and the phylogenetic network showed evident clusters. Thus, most of the worms on each farm were genetically more closely related to each other, and distinct from the worms on other farms. However, each farm contained a few individuals that were more closely related genetically to worms on other farms suggesting new immigrants or retention of polymorphism from the original founders. This may indicate that the investigated farms were colonized by a common genotype in the past and that genetic bottlenecking and subsequent drift have occurred on each farm. This supports the idea that worm eggs deposited by the previous flock infected most laying hens [14]. The alternative, but less likely, explanation is that all of the different farms originally were infected with different genotypes. However, we know of no previous studies on the population genetics of this poultry roundworm based on molecular markers that could be used to test these ideas.

It is unclear how the biological characteristics of a particular parasitic nematode affect the outcome of the genetic variation, especially when studied with a molecular technique targeting dominant multilocus markers, such as with the AFLP. So, our results need to be interpreted with caution. Still, it is interesting to note that the exception to the above pattern is the sample from the Danish farm, where the worms were scattered in three clusters (in the network) associated with different Swedish farms. This suggests the intriguing possibility that the Danish Ascarids may be the historical source of some of the genetic variation among the Swedish Ascarids, which may have occurred in the past. This is a hypothesis that could be tested by collecting similar data from a wider sample of Danish farms.

The estimates of population mutation rate (4N_μ_ = 0.11) and migration rate (N_m_ = 1.7) indicate that the low genetic diversity might be a result of either a low mutation rate (so that new alleles are produced infrequently) or of movement of worms among farms (facilitating gene exchange). Most of the genetic diversity appeared to be under neutral evolution (75% of the fragments), suggesting a lack of strong selection in these nematodes. Geography, parental type and housing conditions apparently did not influence the genetic structure of the worms, suggesting that similar transmission patterns occur irrespective of production system. Unfortunately, no quantitative data are available for direct comparison.

## Conclusions

Our analysis suggests that *A. galli*, which has been recently introduced to the investigated laying hen farms, has undergone a small amount of differentiation due to genetic drift or founder effects, and thus formed genetically distinct but still closely related *A. galli* isolates on the different farms. This conclusion accords with epidemiological data from Jansson et al. [13] and Höglund and Jansson [14], who suggested that laying hens in Sweden are infected with resident *A. galli* eggs that have survived the cleaning procedures of the empty chicken houses between consecutive flocks, and are thus present before the replacement pullets arrive at the farms.

## Competing interests

The authors declare that they have no competing interests.

## Authors’ contributions

JH conceived and coordinated the study, analysed the results and wrote the first draft together with DJ. DM carried out the molecular genetic analyses. AE contributed to the laboratory work and generation of the molecular data. PN critically revised the manuscript and contributed with interpretation of the data. All authors read and approved and contributed to the final version of the manuscript.
